# Body composition during early infancy and its relation with body composition at 4 years of age in Jimma, an Ethiopian prospective cohort study

**DOI:** 10.1038/s41387-018-0056-7

**Published:** 2018-09-07

**Authors:** Bitiya Admassu, Jonathan C. K. Wells, Tsinuel Girma, Tefera Belachew, Christian Ritz, Victor Owino, Mubarek Abera, Rasmus Wibaek, Kim F. Michaelsen, Pernille Kæstel, Henrik Friis, Gregers S. Andersen

**Affiliations:** 1 Department of Population and Family Health, Faculty of Public Health, Jimma University, Jimma, Ethiopia; 2 Department of Nutrition, Exercise and Sports, University of Copenhagen, Copenhagen, Denmark; 3 Childhood Nutrition Research Centre, UCL Great Ormond Street Institute of Child Health, London, UK; 4 Department of Pediatrics and Child Health, Faculty of Medical Sciences, Jimma University, Jimma, Ethiopia; 5 Technical University of Kenya, Haile Selassie Avenue, Nairobi, Kenya; 6 Department of Psychiatry, Faculty of Medical Sciences, Jimma University, Jimma, Ethiopia; 7 Steno Diabetes Center Copenhagen, Gentofte, Denmark

## Abstract

**Background/Objectives:**

Low and high birth weight and rapid weight gain during infancy are associated with childhood obesity. Associations of birth and infancy body composition (BC) growth with childhood BC remain unknown in low-income countries. We aimed to investigate the associations of fat mass (FM) and fat-free mass (FFM) at birth and its accretion during early infancy with FM and FFM at the age of 4 years.

**Methods:**

In the infant Anthropometry and Body Composition (iABC) cohort, BC was assessed at six consecutive time points from birth to 6 months and at 4 years of age by air displacement plethysmography. Multiple linear regression models were used to determine the association between FM and FFM at birth and their accretion rates during infancy and FM index (FMI) and FFM index (FFMI) at 4 years in 314 children.

**Results:**

One kilogram higher FFM at birth was associated with a 1.07 kg/m^2^ higher FFMI (95% CI 0.60, 1.55) at 4 years while a one SD increment in FFM accretion rate from 0 to 6 months was associated with a 0.24 kg/m^2^ increment in FFMI (95% CI 0.11, 0.36) and with a 0.20 kg/m^2^ higher FMI at 4 years (*β* = 0.20; 95% CI 0.04, 0.37). FFM at birth did not predict FMI at 4 years. FM at birth was associated with 1.17 kg/m^2^ higher FMI at 4 years (95% CI 0.13, 2.22) whereas FM accretion from 0 to 4 months was associated with an increase in FMI of 0.30 kg/m^2^ (95% CI 0.12, 0.47). FM at birth did not predict FFMI at 4 years, and neither did FM accretion from 0 to 4 months.

**Conclusions:**

A higher FFM in early infancy predicted higher FFMI at 4 years while a higher FM accretion during early infancy predicted higher FMI at 4 years. Follow-up studies are merited to explore associations of childhood BC with cardio-metabolic risk later in life.

## Introduction

Childhood obesity is a major public health problem worldwide^1^. Its prevalence is dramatically rising^2^ and childhood obesity is an important risk factor for chronic disease occurrence and mortality in adulthood^3,4^.

Numerous studies from high-income countries have shown that birth weight^5–9^ and weight gain during infancy^5–7,10–13^ are positively associated with later adiposity. However, childhood adiposity was typically assessed using body mass index (BMI) or BMI-for-age standard deviation score^6,11^. Although anthropometric measurements such as BMI and skinfold thickness are proxy indicators for body fatness, they are unable to precisely and accurately differentiate variability in fat mass (FM) and fat-free mass (FFM), and it has been suggested that BMI is suboptimal for research investigating childhood obesity^14,15^. Further, most studies on early growth and later obesity focused on weight gain^16^ as the main explanatory variable. Weight gain comprises the accumulation of two types of tissue (FM and FFM), which may have very different associations with later body composition and health. Some studies from high-income countries have associated rapid weight gain during infancy with FM^17–20^ and FFM^17,19^ in childhood. In contrast, the few studies conducted in low- and middle-income countries have associated birth weight and rapid infant weight gain with later lean mass^21–23^ but not with FM, indicating that greater early weight gain is broadly beneficial. It is however not known whether such associations apply to low-income countries that are undergoing nutrition transition^24,25^ and where childhood overweight is an emerging public health problem^26,27^ and co-existing with undernutrition^28^. Such information is crucial in order to promote healthy levels of lean tissue and also to design strategies for preventing excess fat accumulation or childhood obesity. The aim of the present study was therefore to examine the association between FM and FFM at birth and during early infancy with fat mass index (FMI) and fat-free mass index (FFMI) at 4 years of age in a study conducted in Jimma, Ethiopia. We hypothesized that birth body composition (FM and FFM) and body composition gain (FM and FFM accretion rates) during infancy are associated with body composition at 4 years differently.

## Materials and methods

### Study design and subjects

We used data from the infant Anthropometry and Body Composition (iABC) cohort, a prospective birth cohort initially established to generate body composition reference data and further aimed to explore the predictors of early growth and identify short and long-term health consequences of early growth^29^. In brief, apparently healthy neonates with term birth, birth weight ≥ 1500 g, with no congenital malformations and parents living in Jimma Town in Ethiopia were enrolled within 48 h after birth at Jimma University Specialized Hospital (JUSH).

### Data collection and measurements

#### Body composition in early childhood

Within 48 h after birth, weight and body composition were measured using PEA POD, an air displacement plethysmograph (ADP) (COSMED, Rome, Italy). With the same instrument, body composition data at 1.5, 2.5, 3.5, 4.5, and 6 months were also recorded. The PEA POD equipment has been validated against deuterium dilution and the golden standard four-component method in this and other populations and found to be accurate and precise^29,30^. A wig cap was used to reduce the volume of the babies’ hair during volume measurement.

Childhood body composition was measured at 4 years (±3 months), using a BOD POD (COSMED, Rome, Italy) with a customized seat insert for children. The BOD POD also relies on ADP methods to measure body volume and the inbuilt scale is used to measure weight^31^.

ADP, which is a two-component method, allows us to calculate FM and FFM from measurements of total body density (Db) since FM and FFM have distinct densities. Db was calculated from body volume (V) which was predicted using the Lohman equation^32^ and body mass (M), as Db = M/V. Using the body density (Db), percent fat was calculated as suggested by Lohman^32^ as shown below:

% fat = (C1/Db−C2) * 100, where C1 and C2 are constants based on age and sex.

Age and sex-specific equations were used to estimate FM and FFM from measurements of Db obtained from ADP. FM density (D_fm_) was assumed to be constant at 0.901 g/ml, and FFM density (D_ffm_) was assumed to be 1.076 for male and 1.072 for female at 4 years as proposed by Fomon et al.^33^. Using Archimedes principle, fat percentage was derived from Db, allowing FFM to be obtained by subtracting FM from body mass.

The BOD POD was calibrated daily using a phantom volume cylinder of known volume. The assessment procedure was explained to the children and their mothers and the children were asked to wear swimsuit and wig cap as loose clothing affects the volume. The swimsuit and wig cap were provided to the children. Body composition assessment was undertaken by trained research assistants. The BOD POD measurement for children from 2 to 6 years has also been validated against a four-component model and found to be “accurate and precise”^34^.

In order to obtain standardized outcome variables (FM and FFM) at 4 years, we calculated two indices of height-normalized body composition: the FMI, calculated as FM (kg)/height (m)^2^, and the FFMI, calculated as FFM (kg)/height (m)^2^^35,36^.

### Covariates

The new Ballard score was used to estimate gestational age^37^. Length at birth and during infancy was measured in a recumbent position to the nearest 0.1 cm using SECA 416 infantometer (SECA, Hamburg Germany) whereas height was measured in standing position using a SECA 213 portable stadiometer (SECA, Hamburg Germany) at the age of 4 years. Sex of the child, birth order, maternal age, maternal schooling and parental socioeconomic status, and breastfeeding were collected through questionnaires. Socioeconomic questions were assessed to generate a wealth index, which was classified into quintiles.

The scale of a bio-impedance analyser (Tanita 418, Tanita Corp., US) was used to measure maternal weight to the nearest 0.1 kg whereas the SECA 213 portable stadiometer (SECA, Hamburg, Germany) was used to measure height to the nearest 0.1 cm. Maternal anthropometry was measured at birth and at 1.5, 2.5, 3.5, 4.5, and 6 months. Duplicate measurements were taken for length/height, and average values used during analysis.

Breastfeeding status was assessed by questionnaire at 3.5 ± 1 months postpartum, and categorized according to the WHO classification^38^ into exclusive breastfeeding, predominant breastfeeding (the main source of food was breast milk while allowing medicine and giving water and liquid drinks), partial breastfeeding (feeding breast milk together with semi-solid or solid foods), and no breastfeeding.

Height-for-age *z*-score (HAZ) were calculated based on the WHO child growth standard^39^ and categorized as stunted (HAZ < −2) or not stunted (HAZ ≥ −2). A detailed description of the cohort establishment, measurements, and categorizations of variables has previously been described^29,40,41^.

### Statistical analysis

Epidata 3.1 (Odense, Denmark, 2003–2008), R version 3.3.5 (R Development Core Team, Vienna, Austria) and Stata 12.0 (StataCorp, TX, USA) were used for data entry, calculation of tissue accretion rates and outcome analysis respectively. Mean ± SD was used to describe continuous variables while *n* (%) was used for categorical variables. We generated average postnatal maternal BMI using weight and height data collected from 1.5 months to 6 months postpartum since there was a substantial number of missing weight measurements at each visit. The Stata package “Zscore06”^42^ was used to generate HAZ. Comparison between children followed up to 4 years and those lost to follow-up was made using two-sample *t*-tests and Chi-square tests. Simple and multiple linear regression models with robust standard errors were fitted. *P* < 0.05 was considered statistically significant.

Body composition measurements in infancy were obtained at repeated intervals and are likely to be correlated within individuals. In order to separate the effect of each time point on body composition, we generated summary measures of body composition data based on the first 6 months. As described previously^29^, a linear trend was observed for FFM during the first 6 months while FM showed a linear trend only up to 4 months. Therefore, linear mixed models were fitted to derive standardized FM accretion rates from birth to 4 months and standardized FFM accretion rates from birth to 6 months of age for boys and girls separately. Similarly, length accretion rates were derived from birth to 4 and birth to 6 months, respectively, and were used as covariates for FM and FFM accretion rates, respectively. Accretion rates were expressed as SD scores. The construction of standardized accretion rates has been described in detail elsewhere^41^.

Potential confounding variables were identified a priori from the literature^17,23,43–45^ and included in the analysis if they changed the estimate of the main exposure variable: child sex (male, female), birth order (1st, 2nd, and ≥3rd), length at birth (continuous), standardized length accretion during infancy, maternal age (continuous), maternal education (no education, primary, and secondary and above), postnatal maternal BMI (continuous), parental socio-economic status (poorest to richest), breastfeeding at 3.5 ± 1 months postpartum (exclusive breastfeeding, predominant breastfeeding, partial breastfeeding, and no breastfeeding), age at 4 years (continuous), and child stunting at 4 years (stunted or not).

A series of models was developed to explore the association between FM and FFM at birth and FM and FFM accretion rates during early infancy, treated as exposures, and body composition outcomes at 4 years of age. Three separate models were fitted for FM and FFM. Model 1: FFM or FM at birth as main exposure variable adjusted for length at birth, sex of the child, birth order, and child age at 4 years. Model 2: model 1 further adjusted for maternal age, maternal education, parental socio-economic status, and maternal postnatal BMI. Model 3: model 2 with further adjustment for breast-feeding status at 3.5 months postpartum and stunting at 4 years. Similar models were considered for FM and FFM accretion rates during early infancy as main exposure variables. Model 1: contain FM or FFM accretion rate during infancy adjusted for length accretion during respective period, birth order, sex, FM or FFM at birth, and age at 4 years. Model 2: same as model 1 but additionally adjusted for maternal education, maternal age, maternal postnatal BMI, and parental socio-economic status. Model 3: same model as model 2 but additionally adjusted for breastfeeding status at 3.5 months postpartum and stunting at 4 years of age. Visual model checking was carried out using residual plots and normal probability plots. Collinearity was also assessed for each model using the variance inflation factor.

Prior to examining the main exposure variables, we explored the association of birth weight with body composition at the age of 4 years by excluding body composition at birth. The association of birth weight was explored to see if we could replicate the finding observed from previous literatures.

## Results

**Fig. 1:**
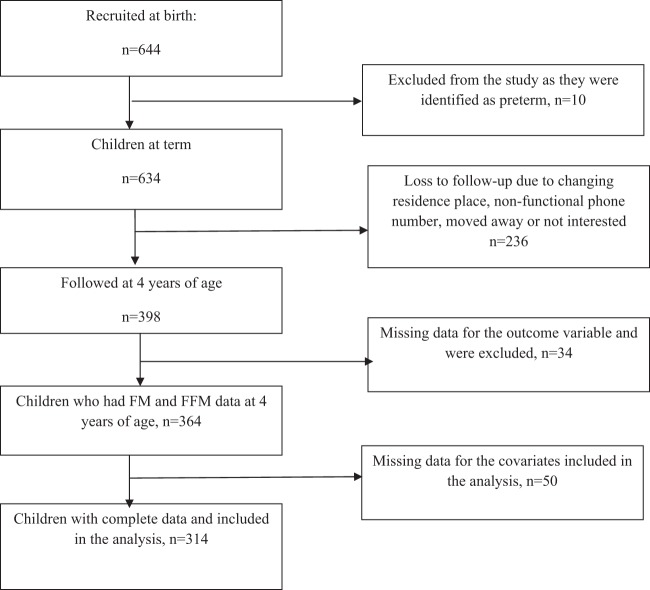
Flow chart showing participants included in the study

Out of 644 children recruited at birth, ten children were found to be preterm (<37 weeks) and were excluded from the study. Of the 634 remaining, 398 (62.8%) were followed up at 48 (±3) months of age, and of these, 364 (91.5%) had body composition data at 4 years. Thus, a total of 314 children with complete data for all the covariates were included in models using FM or FFM at birth as main exposure variables (Fig. 1), whereas 307 children were included in models using FM or FFM accretion rates as main exposure variables. Baseline characteristics were not markedly different between children with and without body composition data at 4 years except that children lost to follow-up were more likely to be first born and from poor families (Supplemental Table 1).

The mean ± SD of FM and FFM at birth was 0.2 ± 0.2 and 2.8 ± 0.3 kg, respectively while mean ± SD of FM and FFM at 4 years were 3.8 ± 1.4 and 10.9 ± 1.2 kg, respectively. The mean ± SD maternal age at birth was 24.7 ± 4.7 years. Around 62% of the mothers had attended primary education (Table 1).

**Table 1:** Description of children and mothers in the iABC cohort study (*n* = 314)

**Birth characteristics** ^**a**^	
Fat-free mass, kg	2.8 ± 0.3
Fat mass, kg	0.2 ± 0.2
Weight, kg	3.1 ± 0.4
Length, cm	49.2 ± 1.9
Male sex, *n* (%)	164 (52.2)
Birth order, *n* (%)	
1st	152 (48.4)
2nd	82 (26.1)
≥3rd	80 (25.5)
Maternal age at delivery, years	24.8 ± 4.6
Maternal education, *n* (%)	
No school	19 (6.1)
Primary	195 (62.1)
≥Secondary	100 (31.8)
**Postnatal characteristics 0**–**4 years**	
Postnatal maternal BMI, kg/m^2^	22.3 ± 3.5
Breastfeeding status at 3.5 months, *n* (%)	
Exclusive	74 (23.6)
Predominant	127 (40.4)
Partial	103 (32.8)
No breastfeeding	10 (3.2)
FM accretion from 0 to 4 months, SD score	−0.03 ± 1.0
FFM accretion from 0 to 6 months, SD score	0.02 ± 1.1
**Child characteristics at 4 years**	
Child age, months	48.3 ± 0.8
Fat-free mass, kg	10.9 ± 1.2
Fat mass, kg	3.8 ± 1.4
Stunted (HAZ < −2), *n* (%)	71 (22.6)

*iABC* infant Anthropometry and Body Composition

Data are shown as mean ± SD unless otherwise stated

^a^Data were obtained within 48 h after delivery

FFM at birth was positively associated with FFMI at 4 years of age, with very similar estimated slope coefficients in all models (Table 2): a 1 kg higher FFM at birth was associated with a 1.07 kg/m^2^ (95% CI 0.60, 1.55, model 3) higher FFMI at 4 years in the fully adjusted model. However, FM at birth showed no association with FFMI in any of the models (Table 2). FM at birth showed positive association with FMI at 4 years in all models. The estimate slope coefficient attenuated from model 1 to model 2 but was relatively similar between models 2 and 3. A 1 kg higher FM was associated with a 1.17 kg/m^2^ (95% CI 0.13, 2.22; model 3) higher FMI at 4 years. In contrast, FFM at birth did not correlate with FMI at 4 years (Table 2).

**Table 2:** Association of fat-free mass (FFM) or fat mass (FM) at birth with fat-free mass index (FFMI, kg/m^2^) and fat mass index (FMI, kg/m^2^) at 4 years of age in the iABC cohort study (*n* = 314)

Birth body composition	Model 1^a^	Model 2^b^	Model 3^c^
**FFMI (kg/m** ^**2**^ **) at 4 years**			
FFM (kg)	1.02 (0.54, 1.49)***	1.10 (0.62, 1.58)***	1.07 (0.60, 1.55)***
FM (kg)	0.08 (−0.65, 0.80)	0.14 (−1.59, 0.87)	0.05 (−0.69, 0.78)
**FMI (kg/m** ^**2**^ **) at 4 years**			
FFM (kg)	0.48 (−0.23, 1.19)	0.44 (−0.26, 1.15)	0.41 (−0.29, 1.11)
FM (kg)	1.40 (0.40, 2.41)**	1.18 (0.16, 2.20)*	1.17 (0.13, 2.22)*

Each row shows separate multiple linear regression result for both FM and FFMI as an outcome. Slope estimates (*β*) shown with 95% confidence interval

^a^Model 1: accretion rates (FFM or FM) adjusting for length at birth, sex of the child, birth order, and child age at 4 years

^b^Model 2: as model 1, further adjusting for parental characteristics (baseline maternal age, maternal education, parental wealth index, and maternal postnatal BMI) measured at 1.5–6 months postpartum

^c^Model 3: as model 2, further adjusting for breastfeeding at 3.5 (±1 months) postpartum and stunted (yes/no) at 4 years

*p < 0.05, **p < 0.01, ***p < 0.001

FFM accretion from 0 to 6 months was positively correlated with FFMI at 4 years in all models, with similar estimated slope coefficients in models 1 and 2 but a slightly higher estimate in model 3. In the fully adjusted model, a one SD increment in FFM accretion from 0 to 6 months was associated with a 0.24 kg/m^2^ increment of FFMI at 4 years (95% CI 0.11, 0.36; model 3). FM accretion during the first 4 months was not associated with FFMI at the age of 4 years (Table 3). FM accretion during the first 4 months was positively associated with FMI at 4 years in all models with very similar estimate. A one SD higher in FM accretion from 0 to 4 months led to a 0.30 kg/m^2^ (95% CI 0.12, 0.47; model 3) higher FMI at 4 years. FFM accretion during the first 6 months was positively correlated with a 0.20 kg/m^2^ (95% CI 0.04, 0.37; model 3) higher FMI at 4 years (Table 3).

**Table 3:** Association of early infancy standardized tissue accretion rates with fat-free mass index (FFMI, kg/m^2^) and fat mass index (FMI, kg/m^2^) at 4 years of age in the iABC cohort study (*n* = 307)

Tissue accretion rate	Model 1^a^	Model 2^b^	Model 3^c^
**FFMI (kg/m** ^**2**^ **) at 4 years**			
FFM (0–6 months)	0.20 (0.08, 0.33)**	0.21 (0.09, 0.33)***	0.24 (0.11, 0.36)***
FM (0–4 months)	−0.02 (−0.15, 0.11)	−0.01 (−0.14, 0.12)	−0.01 (−0.14, 0.12)
**FMI (kg/m** ^**2**^ **) at 4 years**			
FFM (0–6 months)	0.21 (0.04, 0.38)*	0.19 (0.03, 0.35)*	0.20 (0.04, 0.37)*
FM (0–4 months)	0.29 (0.11, 0.47)**	0.30 (0.12, 0.48)***	0.30 (0.12, 0.47)***

Each row shows separate multiple linear regression result for the outcome (FM and FFMI). Slope estimates (*β*) shown with 95% confidence interval

^a^Model 1: accretion rates (FFM or FM accretion rates) adjusted for length accretion rate, sex of the child, birth order, FFM or FM at birth, and child age at 4 years

^b^Model 2: as model 1, further adjusting for parental characteristics (baseline maternal age, maternal education, parental wealth index, and maternal postnatal BMI) measured at 1.5–6 months postpartum

^c^Model 3: as model 2, further adjusting for breastfeeding at 3.5 (±1 months) postpartum and stunted (yes/no) at 4 years

*p < 0.05, **p < 0.01, ***p < 0.001

Birth weight was associated with both FMI and FFMI at 4 years of age and had a stronger association with FFMI (*β* = 0.75; 95% CI 0.33, 1.16; model 3) at 4 years compared to FMI (*β* = 0.66; 95% CI 0.07, 1.26; model 3) (result not shown).

## Discussion

This study showed that FFM at birth was positively associated with FFMI at 4 years while FM at birth correlated positively with FMI at 4 years. The postnatal FFM accretion rate predicted both FFMI and FMI positively while FM accretion during the first 4 months predicted only FMI at 4 years, and not FFMI. Previous studies have identified higher fetal growth and growth during infancy in terms of weight at birth and weight change during infancy linked with higher lean mass, FM, and indicators of childhood obesity. However, the current study broke new ground in distinguishing the associations of FM and FFM accretion during early life with body composition during childhood.

We found a positive association between FFM at birth and FFMI at 4 years independent of length at birth, maternal BMI, and other potential confounders. This supports the hypothesis of “fetal programming” of later health, which may reflect the tracking of fetal FFM into the childhood period. In the same cohort, we have previously shown that FFM at birth was a positive predictor of height^46^ and cognitive development at 2 years^47^ and linear growth from 1 to 5 years of age^41^. Collectively, these results indicate that higher prenatal growth, if mainly driven by lean mass, benefits health and development during childhood. Greater FFM at birth may indicate better quality of maternal nutritional intake during pregnancy and healthier intrauterine experience of the fetus. In relation to this, a prospective study from Colorado, USA revealed that higher maternal consumption of solid fat, whole grain, fruits, cheese, poultry, nuts, and sugar during pregnancy predicted higher FFM at birth^48^. Another prospective study from the Netherlands showed that maternal protein intake (both animal and plant sources) during pregnancy predicted FFM but not FM at 6 years of age^49^.

In the current study, FM at birth showed a positive association with FMI at 4 years in all models. FM accounts for a small proportion of the total birth weight in the study population. Though fat stores are utilized to varying degrees to fund immune function and may promote survival during infant and early childhood malnutrition^50^, our study of a healthy population revealed that greater birth fat relates to higher level of FM at 4 years of age. This finding indicates tracking of prenatal FM to the childhood period. In the same cohort, we have previously shown that FM at birth positively predicted higher social and emotional problems at 5 years of age^51^, and that it did not correlate with height^46^ or child development^47^ at 2 years, or with linear growth from 1 to 5 years^41^. Together, these findings indicate that storing fat in prenatal life to protect against post-natal malnutrition comes at a cost to growth and cognitive development.

This study also showed that FFM accretion from 0 to 6 months positively predicted FFMI at the age of 4 years independent of potential confounders. Since FFMI adjusts FFM for height, this indicates that infant FFM accretion promotes childhood FFM beyond any association with linear growth. A possible explanation could be the insulin-like growth factor 1 secretion that stimulates protein accretion, which is known to contribute to the development of muscle mass^52^.

Further, nutritional intake during the postnatal period is the main environmental factor associated with body composition changes during infancy. However, if the infant loses appetite due to illness or infection, low food intake results in lower FM and FFM gain in infancy and infection also costs calories. Thus, infancy FFM accretion might mediate the link between nutrition intakes during infancy with childhood FFM.

FFM accretion during the first 6 months showed a positive association with FMI. We have proposed previously that to endure a given duration of undernutrition, a child with larger FFM will require larger amounts of energy and hence store larger fat stores^53^. All other things being equal, larger FFM during infancy is associated with larger FM in childhood. Thus, a baby with more FFM in infancy tends to become a child with more FFM (Table 3), which then favors more FM. We therefore speculate that the observed association might indicate that early infancy FFM tracked into childhood, thereby influencing the level of fat storage at the same time.

Postnatal FM accretion from 0 to 4 months was positively associated with FMI at 4 years. Several studies from high-income settings^17,20,54^ have linked infant weight gain or fat accretion with higher levels of childhood adiposity, and one study in Brazil^55^ showed consistent results. However, a few studies from low- and middle-income countries showed opposite association^21–23^ in which weight gain associated with FFM but not with FM. Contrasting findings from previous studies conducted in high- and middle-income countries regarding the relation between infancy weight gain and body composition during childhood might be due to lack of data on early life body composition, and hence the possibility to differentiate FM and FFM. Additionally, it may be due to differences in the populations studied, while within the same population, different patterns of infant fat accretion might promote different patterns of childhood body composition^56^. The association between weight gain during infancy and FMI in childhood reported by other studies, might be explained by FM accretion during the postnatal period. Thus, in terms of FM accretion, both prenatal as well as postnatal period may be required to acquire higher FMI at 4 years.

This study therefore breaks new ground in explaining the possible mechanism by which early life weight gain relates to childhood fatness. In our previous analyses, postnatal FM accretion had a positive association with linear growth from 1 to 5 years^41^. However, studies conducted in middle^57^- and high^58^- income countries have found that infancy and childhood FM is associated with markers of cardio-metabolic status in children and adolescents. Further studies from the current and similar cohorts are needed to explore if this pattern exists in children from low-income countries.

Birth weight was a positive predictor of both FMI and FFMI at 4 years which is in line with some previous studies^17,59^, while other studies found an association between birth weight and FFM only^23,60^. However, since birth weight is the sum of both FM and FFM, it might not be a good indicator. Thus, our study demonstrates the importance of studying infancy body composition for designing specific interventions to prevent excess fatness and maintain lean mass during childhood.

### Strengths and limitations

We used repeated body composition measurements during infancy from ADP, which is an accurate and precise method to measure tissue accretion^30^. FM and FFM accretion during early infancy were predicted based on available data points for those children who had body composition data at birth and at one or more times in any of the follow-up visits. This is a useful approach to predict accretion rates for children with a minimum of two measurements. In addition, the outcome was also measured using ADP rather than simple anthropometry. Our findings are based on multivariate analysis with a priori identified predictors.

Though this study has a number of strengths, it is not free from limitations that could affect the interpretation.

The body volume estimation done during the BOD POD assessment might be affected by measurement error as lung volume might be inaccurate due to improper position, uncovering of the hair or children crying. However, we minimized measurement error using the instrumentation’s pediatric protocol, covering the child’s hair with a cap, and repeating measurements when a child cried. Further, we did not collect data on pre-pregnancy weight, pregnancy weight gain, and dietary intake during pregnancy, which might all contribute to birth body composition and confound the associations. In addition, we did not assess the physical activity of children. We did not adjust the analysis for time of solid food introduction, childhood feeding pattern, or morbidity history, which might confound the current findings. However, the studied infants were urban-living, generally healthy and infants with serious medical conditions at birth were not recruited for the study but appointed for the neonatology unit at the hospital. Loss to follow-up might also affect the interpretation of the findings: in particular, the observed differences in birth order and wealth index could lead to a slight bias in estimated associations. Exclusion of children from analyses due to missing covariate information, which occurred due to many different reasons, most likely introduced a negligible bias.

## Conclusions

This study provided a more comprehensive analysis of the relation between components of weight in early infancy with childhood body composition than what was possible from weight gain alone. A higher FFM at birth and during early infancy predicted higher FFMI at 4 years. A higher FM accretion at birth and during the first 4 months predicted higher FMI at 4 years. Our findings provide support to identification and promotion of optimal maternal nutrition and infant feeding practices that help to maintain higher lean tissue and prevent excess fat storage during infancy and childhood. Further follow-up of the cohort may reveal whether these associations track into later childhood and adolescence.

## Electronic supplementary material

SUPPLEMENTAL TABLE 1
