# Supplementary material for: Body composition during early infancy and its relation with body composition at 4 years of age in Jimma, an Ethiopian prospective cohort study
Source: Nutr Diabetes. 2018 Sep 7;8:46. doi: 10.1038/s41387-018-0056-7 (PMC6127223; doi:10.1038/s41387-018-0056-7)
Supplement: Supplementary file 1 — SUPPLEMENTAL TABLE 1 [file 41387_2018_56_MOESM1_ESM.doc]

| **SUPPLEMENTAL TABLE 1** Comparison between children with and without body composition data at 4 years of age in the iABC cohort ^1^ | | | |
| --- | --- | --- | --- |
|  | Had body composition data at 4 years (*n*=364) | Had no body composition data at 4 years (*n*=270) | *P* value |
| Birth characteristics^2^ |  |  |  |
| Length, cm | 49.2 **±** 1.9 | 49.0 **±** 2.1 | 0.29 |
| FFM, kg | 2.8 **±** 0.3 | 2.8 **±** 0.4 | 0.21 |
| FM, kg | 0.2 **±** 0.2 | 0.2 **±** 0.2 | 0.55 |
| Birth weight, kg | 3.0 **±** 0.4 | 3.0 **±** 0.4 | 0.23 |
| Female sex, (%) | 178 (48.9) | 143 (52.9) | 0.34 |
| Birth order, (%) |  |  | 0.01 |
| 1^st^ | 164 (45.7) | 150 (57.7) |  |
| 2^nd^ | 97 (27.0) | 63 (24.2) |  |
| ≥ 3^rd^ | 98 (27.3) | 47 (18.1) |  |
| Maternal education, (%) |  |  | 0.89 |
| None | 44 (7.0) | 20 (7.6) |  |
| Primary | 381 (60.9) | 159 (60.2) |  |
| ≥Secondary | 201 (32.1) | 85 (32.2) |  |
| Parental wealth index, (%) |  |  | 0.01 |
| 1^st^ (poorest) | 67 (18.7) | 73 (28.6) |  |
| 2^nd^ | 85 (23.7) | 49 (19.2) |  |
| 3^rd^ | 90 (25.1) | 59 (23.1) |  |
| 4^th^ | 71 (19.8) | 34 (13.3) |  |
| 5^th^ (richest) | 46 (12.8) | 40 (15.7) |  |
| ^1^ FFM; fat-free mass, FM; fat mass, iABC; infant Anthropometry and Body Composition.  ^2^ Data were obtained within 48 hours after delivery. Groups were compared using two-sample t-tests and chi-square tests as appropriate. | | | |
